# Bioinspired Nanocomposite for Targeted Immunoengineering and Improved Tendon Regeneration

**DOI:** 10.34133/cbsystems.0503

**Published:** 2026-04-23

**Authors:** Xianfeng Wang, Zhihong Xu, Yuwen Shangguan, Biyong Deng, Hao Nan, Zhecheng Jiang, Huan Li, Litao Yan

**Affiliations:** ^1^ Department of Orthopedic Surgery, Beijing Jishuitan Hospital Guizhou Hospital, Guiyang 550014, China.; ^2^ Division of Sports Medicine and Adult Reconstructive Surgery, Department of Orthopedic Surgery, Nanjing Drum Tower Hospital, The Affiliated Hospital of Nanjing University Medical School, Nanjing University, Nanjing 210008, China.; ^3^ Department of Orthopaedics, Changzhou Maternal and Child Health Care Hospital, Changzhou Medical Center, Nanjing Medical University, Changzhou 213003, China.; ^4^ Department of Articular Orthopaedics, The First People’s Hospital of Changzhou, The Third Affiliated Hospital of Soochow University, Changzhou 213003, China.

## Abstract

Tendon healing is impaired by excessive reactive oxygen species, inflammatory cell infiltration, and insufficient osteogenic differentiation during treatment. Therefore, simultaneously modulating the local inflammatory microenvironment and enhancing osteogenic regeneration are critical for effective tendon repair. Here, we designed a biomimetic nanoparticle system, macrophage-membrane-coated Prussian blue@kartogenin (KGN@PB@CM), loaded into a Pluronic@F127/hyaluronic acid (HA-F127) thermosensitive hydrogel. The HA-F127 hydrogel provides excellent hydrophilicity and enables sustained release of M-PB@KGN nanoparticles. Leveraging homologous targeting via the cell membrane, the KGN@PB@CM nanoparticles are efficiently delivered to macrophages, promoting their polarization toward a prohealing phenotype. Concurrently, KGN enhances the migration and differentiation of stem cells. Overall, we propose a “dual-modulation” strategy (anti-inflammatory and pro-osteogenic differentiation) to synergistically accelerate tendon healing.

## Introduction

Tendon injuries are among the most prevalent musculoskeletal disorders, often resulting from sport-related trauma, degenerative diseases, or aging [1,2]. Despite their high incidence, tendon healing remains a great clinical challenge due to the tissue’s poor regenerative capacity and the formation of fibrotic scars, which compromise mechanical functionality [3]. Current therapeutic strategies, including surgical repair, physical therapy, and anti-inflammatory medications, often fail to restore the original biomechanical properties of tendons [4]. Moreover, excessive reactive oxygen species (ROS) accumulation, persistent inflammatory infiltration, and insufficient tenogenic differentiation further impair the healing process [5]. Thus, there is an urgent need for innovative approaches that can simultaneously modulate the inflammatory microenvironment and enhance tenocyte regeneration to achieve functional tendon repair.

Effective tendon regeneration requires a balanced immune response and proper stem cell differentiation [6,7]. During the early inflammatory phase, excessive ROS and proinflammatory M1 macrophage polarization exacerbate tissue damage, while inadequate transition to the reparative M2 phenotype impedes matrix remodeling [8–10]. Therefore, strategies that scavenge ROS and promote macrophage polarization toward the M2 phenotype are critical for mitigating inflammation. In addition, stem cells play a pivotal role in tendon repair, but their therapeutic potential is often limited by poor recruitment and inefficient tenogenic differentiation. Kartogenin (KGN), a small-molecule drug, has shown promise in promoting stem cell migration and tenocyte differentiation by up-regulating tendon-related markers [11–13]. However, its clinical translation is limited by rapid clearance and lack of targeted delivery [14,15]. To address these challenges, nanotechnology-based delivery systems have emerged as a powerful tool to enhance drug stability, enable controlled release, and improve bioavailability.

In current investigation, we developed a biomimetic nanoparticle (NP) system composed of macrophage-membrane-coated Prussian blue@KGN (KGN@PB@CM) loaded into a thermosensitive Pluronic@F127/hyaluronic acid (HA-F127) hydrogel for synergistic tendon healing. The HA-F127 hydrogel provides a hydrophilic microenvironment for sustained drug release, while the macrophage membrane enables homologous targeting to inflammatory sites, enhancing NP uptake by macrophages [16]. Prussian blue (PB), a potent ROS scavenger, mitigates oxidative stress and promotes M2 macrophage polarization, thereby resolving inflammation. Concurrently, KGN is released to recruit stem cells and drive tenogenic differentiation, addressing the limitation of insufficient cell regeneration [17,18]. In a murine tendon injury model, this dual-functional “anti-inflammatory and protenogenic” strategy demonstrated accelerated healing, reduced fibrosis, and improved biomechanical properties. Our conclusions identify the potential of this nanocomposite hydrogel as a promising treatment platform for functional tendon repair.

## Methods

### Preparation of PB nanozymes

Polyvinyl pyrrolidone (PVP)-coated PB nanozymes were synthesized through a hydrothermal approach. Specifically, 3 g of PVP and 131.9 mg of K_3_[Fe(CN)_6_]·3H_2_O were precisely weighed and dissolved separately at RT in 40 ml of 0.01 M HCl solution under continuous magnetic stirring into complete dissolution. The mixed solution was placed in a 3-neck flask and heated in an 80 °C water bath for 20 h. The product was centrifuged to remove unreacted precursors after natural cooling to RT. The precipitate was subsequently washed several times with deionized water and absolute ethanol to obtain purified PB nanozymes.

### Preparation of KGN@PB nanozymes

The KGN-loaded PB NPs (KGN@PB NPs) were prepared as follows: PB NPs were first dispersed in distilled water. Subsequently, 0.0315 ml of dimethyl sulfoxide solution containing 1 mg of KGN (100 mM) was dropwise added to 100 μl of PB NPs solution (1 mg) under light-protected conditions. The mixture was allowed to react for 24 h with constant stirring at 1,000 rpm, followed by centrifugation at 13,000 rpm for 15 min to acquire KGN@PB NPs. The resulting NPs were then washed with distilled water to remove unbound components.

### Extraction of macrophage membranes

The harvested macrophages were resuspended in phosphate-buffered saline (PBS) and immediately stored at −80 °C. The frozen cells were subjected to 2 to 3 freeze-thaw cycles until a viscous, mucus-like consistency was achieved. The resulting lysate was transferred to a penicillin vial, mixed with a minimal volume of PBS, and sonicated at the lowest power setting (10%) using a pulsed mode (1-s on/1-s off for 2 min). The sonicated suspension was put into centrifugation at 3,000 to 4,000*g* for 10 min to get rid of cell debris and organelles, retaining the supernatant. For membrane purification, the supernatant was subjected to high-speed centrifugation at 14,800*g* (4 °C for 30 min), followed by a 10-min rest and an additional 30-min centrifugation. Alternatively, ultracentrifugation at 100,000*g* for 30 min could be used. After resuspension in prechilled PBS, the final membrane pellet was homogenized by gentle pipetting.

### Preparation of KGN@PB@CM nanozymes

The macrophage membrane fragments were mixed with KGN@PB NPs at a mass ratio of 1 mg (NPs):200 μg (CM) in PBS. The mixture was subjected to intermittent sonication (25% power, 3-s on/6-s off) for 10 min to facilitate membrane fusion. Subsequently, the sample was given centrifugation at 12,000 rpm (4 °C for 10 min) to take off unbound membrane components, yielding the final KGN@PB@CM NPs.

### Preparation of KGN@PB@CM-loaded gel

Pluronic F127/HA solutions were prepared in ultrapure water at a fixed concentration of 24% (w/v). The solutions were equilibrated at either RT or 37 °C to evaluate thermoresponsive behavior. Gelation kinetics were monitored through visual observation of solution turbidity changes. For KGN@PB@CM loading, CM@KGN@PB was physically mixed with HA-F127 solutions.

### DPPH· scavenging assays

The preparation of DPPH working solution was processed by dissolving 1 mg of DPPH in 24 ml of absolute ethanol, followed by 5 min of sonication for complete dissolution. A 1-ml aliquot was then diluted with ethanol to adjust the absorbance to 0.6 to 1.0 at 519 nm, as measured by UV-Vis spectroscopy. For antioxidant activity evaluation, 1 ml of the DPPH working solution was incubated with PB, KGN@PB, or CM@KGN@PB nanozymes for 30 min under dark conditions to prevent photodegradation. The radical scavenging capacity was subsequently measured at 519-nm absorbance via UV-Vis spectroscopy.

### Cell culture

Primary rat TDSCs were separated from the Achilles tendons of male Sprague–Dawley rats (8 weeks old), following previously established protocols with minor modifications. Briefly, harvested tendons were minced into ~1-mm^3^ fragments and subjected to enzymatic digestion using 0.1% collagenase type I and dispase (2.5 U/ml; Gibco) in a shaking incubator at 37 °C for 2 h. The resultant cell suspension was filtered through a 70-μm strainer, centrifuged, and resuspended in α-minimum essential medium (Gibco) supplemented with 10% fetal bovine serum (FBS; Gibco) and 1% penicillin–streptomycin. Cells were cultivated at a humidified atmosphere with 5% CO_2_ at 37 °C, with passages 2 to 4 utilized for all subsequent experiments. The macrophage cell line RAW 264.7 (American Type Culture Collection [ATCC], TIB-71) was maintained in Dulbecco’s modified Eagle’s medium (Gibco) containing 10% FBS and 1% penicillin–streptomycin under 37 °C and 5% CO_2_ culturing conditions. Cells were passaged every 2 to 3 d and utilized consistently between passages 3 and 10.

### Biocompatibility assay in vitro

To assess the cytocompatibility of the KGN@PB@CM composite, we systematically evaluated the viability, proliferation, and oxidative stress resistance of TDSCs using a combination of live/dead staining, EdU incorporation, CCK-8 assay, and ROS detection.

For live/dead staining, TDSCs were seeded in 24-well plates and incubated with KGN@PB@CM for 24 h at a density of 2 × 10^4^ cells per well. Following treatment, cells were stained using calcein-AM/PI reagents (Beyotime, China) following the manufacturer’s protocol. Live cells emitted green fluorescence, while dead cells appeared red. Fluorescence images were captured with a Leica epifluorescence microscope (Leica, Germany), and viability was quantified via ImageJ software.

The assessment of cell proliferation was used by the CCK-8 (Dojindo, Japan). TDSCs were seeded in 24-well plates and incubated with KGN@PB@CM for 24, 48, and 72 h at 2 × 10^4^ cells per well. After incubation, 10% CCK-8 reagent was supplement to each well and kept at 37 °C for 2 h. The determination of absorbance was processed at 450 nm utilizing a microplate reader (Thermo Fisher Scientific, USA).

EdU incorporation assays were used to further quantify proliferative activity. TDSCs were incubated with 10 μM EdU (Beyotime, China) for 3 h after treatment with KGN@PB@CM. The process involved fixing, permeabilizing, and staining the cells following the manufacturer’s instructions. Fluorescence images were acquired, and EdU-positive cells were quantified using ImageJ.

To evaluate the antioxidative capacity of the material, 200 μM hydrogen peroxide (H_2_O_2_) treatment was given within TDSCs environment for 6 h to induce ROS generation, with or without KGN@PB@CM. Intracellular ROS levels were identified via DCFH-DA fluorescent probes (Beyotime, China), and fluorescence intensity was assessed microscopically and quantitatively utilizing ImageJ.

### Macrophage polarization assays

RAW 264.7 macrophages (ATCC, USA) were seeded into 6-well plates and incubated with KGN@PB@CM extract (0.1 g/ml) for 72 h at a density of 1 × 10^5^ cells per well. To mimic a proinflammatory microenvironment and elicit M1 polarization, LPS (100 ng/ml) was administered to cells during the terminal 24-h incubation period. Experimental groups included untreated controls, LPS-treated controls, PB+LPS, KGN@PB+LPS, and KGN@PB@CM+LPS groups.

### Immunofluorescence staining

After treatment, macrophages were put into fix with 4% paraformaldehyde for 15 min, permeabilized with 0.1% Triton X-100 for 10 min, and blocked with 5% bovine serum albumin (BSA) for 1 h at RT. Cells grew overnight at 4 °C within primary antibodies against iNOS (M1 marker), CD86 (M1 marker), and CD206 (M2 marker) (Abcam, UK; 1:500). After washing, Alexa Fluor 488- or 594-conjugated secondary antibodies (Invitrogen, USA) were applied for 1 h at RT. Cell nuclei were stained with 4′,6-diamidino-2-phenylindole (DAPI). Images were captured by a Leica fluorescence microscope and analyzed via ImageJ software.

### Quantitative real-time PCR

The extraction of total RNA was performed by RAW 264.7 cells utilizing TRIzol reagent (Invitrogen, USA), and reverse transcription was used via a cDNA synthesis kit (Takara, Japan). qRT-PCR was processed via SYBR Green Master Mix (Bio-Rad, USA) on a StepOnePlus Real-Time PCR System (Applied Biosystems, USA). Primer sequences targeting proinflammatory markers (CD86, IL-6, TNF-α, and iNOS) and anti-inflammatory markers (CD206, Arg-1, and IL-10) were designed and synthesized by ServiceBio (Wuhan, China). See Table 1 for all primer sequences. Normalization to glyceraldehyde-3-phosphate dehydrogenase (GAPDH) was conducted for relative gene expression levels that were calculated by the 2^−ΔΔCt^ method.

**Table 1.** Primer sequences for qPCR

Gene	Forward primer (5′→3′)	Reverse primer (5′→3′)
Col1a1	GAGCGGAGAGTACTGGATCG	GTTGGGATGGAGGGAGTTTA
Tnmd	AGCAGCCATCAAAGGTGGAG	AGTTGGGATGCTCTGTGCTG
Mkx	CTGAGGAGGATGAGCGTCAA	GGGACTGTTGCTCCTCTTGA
Scx	CAGTGGCTGCTGAAGTTCTG	TGCTTCTGCTTTGTCTTTGG
iNOS	CAGCTGGGCTGTACAAACCTT	CATTGGAAGTGAAGCGTTTCG
Arg-1	CTCCAAGCCAAAGTCCTTAGAG	AGGAGCTGTCATTAGGGACATC
TNF-α	CCCTCACACTCAGATCATCTTCT	GCTACGACGTGGGCTACAG
IL-6	TAGTCCTTCCTACCCCAATTTCC	TTGGTCCTTAGCCACTCCTTC
IL-10	GCTCTTACTGACTGGCATGAG	CGCAGCTCTAGGAGCATGTG
CD206	TGTGGGTTGCTATCACTCTCTATG	CTCAGTCTGTTTTGGGCTTGTC
GAPDH	AGGTCGGTGTGAACGGATTTG	TGTAGACCATGTAGTTGAGGTCA

### ELISA assay

Cell culture supernatants were obtained after treatment and maintained at −80 °C until analysis. IL-1β, IL-6, TNF-α, IL-10, and TGF-β1 levels were assessed by ELISA kits (MULTI SCIENCES, China) following the manufacturer’s instructions. A microplate reader (Thermo Fisher Scientific, USA) was utilized to record absorbance at 450 nm.

### In vivo animal study

The Institutional Animal Care and Use Committee of Beijing Jishuitan Hospital Guizhou Hospital granted approval for all animal experiments, which were then conducted in compliance with the Basel Declaration guidelines (approval number: LW20250301).

### Rat Achilles tendon injury model and surgical treatment

The study involved randomly assigning male Sprague–Dawley rats, aged 8 to 10 weeks and weighing 220 to 250 g, into 4 groups (*n* = 20 per group): sham, model, NC-gel, and NPs@gel. The Achilles tendon injury model was established by performing a full-thickness transverse tenotomy at the mid-substance of the right Achilles tendon under isoflurane anesthesia. For the sham group, only skin incision was conducted without tendon injury or repair. For the model group, the tendon was transected and immediately repaired using a modified Kessler suture without any additional treatment. In the NC-gel and NPs@gel groups, the tendon was wrapped with NC-gel or KGN@PB@CM-loaded hydrogel (NPs@gel), respectively, after suturing. Skin was closed with interrupted sutures, and meloxicam (3 mg/kg, intraperitoneally) was administered postoperatively to relieve pain.

### Histological evaluation of tendon healing

To assess tissue morphology and collagen deposition, Achilles tendon samples were harvested at 2 and 4 weeks postsurgery from rats in the sham, model, NC-gel, and NPs@gel groups (*n* = 5 per group). After fixation in 4% paraformaldehyde for 24 h, specimens were dehydrated in 30% sucrose, embedded in optimal cutting temperature compound (OCT), and longitudinally sectioned into 10-μm slices.

During H&E staining, standard protocols were applied to visualize cellular infiltration, vascularization, and general tissue architecture. For Masson’s trichrome staining, sections were processed using a commercial staining kit (ServiceBio, China) following the manufacturer’s instructions to evaluate collagen fiber density and organization. Collagen fibers, nuclei, and cytoplasm were stained blue, brown or black, and red, respectively.

All sections were imaged by a bright-field microscope (Leica, Germany), and semiquantitative analysis of collagen alignment and tissue cellularity was performed using ImageJ software with a blinded protocol. Quantitative histological analysis was performed using the Bonar scoring system, as previously described [19].

### Immunofluorescence staining of tendon tissue

At postoperative weeks 2 and 4, animals were euthanized, and Achilles tendon samples were harvested within 4% paraformaldehyde overnight and then cryoprotected in 30% sucrose. The embedding of samples in OCT compound was followed by longitudinal sectioning (10 μm). Sections were blocked with 5% BSA and fixed with primary antibodies targeting CD86, CD206, TNMD, COL1, and α-SMA overnight at 4 °C, followed by fluorescent secondary antibodies and DAPI counterstaining. Images were captured using a Leica epifluorescence microscope and addressed by ImageJ software for fluorescence quantification.

### Gait functional evaluation (CatWalk analysis)

To evaluate functional recovery, rats were subjected to gait analysis via the CatWalk XT system (Noldus) at weeks 2 and 4. Each rat was allowed to freely traverse the glass walkway, and 3 valid runs were collected per animal. Key gait parameters, including print area, max contact area, duty cycle, and swing speed, were extracted using automated software and statistically compared across groups.

### Biomechanical testing of repaired tendons

At 8 weeks postsurgery, Achilles tendon–bone complexes were dissected and subjected to tensile biomechanical testing via a universal testing machine (Instron 5944). After preloading at 0.1 N, tendons were elongated at a constant rate of 5 mm/min until failure. The records of load–displacement curve determined ultimate tensile strength, stiffness, and Young’s modulus.

### In vivo biosafety evaluation

To evaluate systemic biosafety, major organs encompassing the heart, lung, spleen, liver, and kidney were harvested from rats at 8 weeks postoperatively (*n* = 5 per group) following treatments with sham, model, NC-gel, and NPs@gel. Samples were preserved in 4% paraformaldehyde, embedded in paraffin, and sliced into 5-μm-thick sections for H&E staining. Morphological assessment was performed under a light microscope (Leica, Germany) to detect signs of inflammation, necrosis, or structural damage.

### Statistical analysis

Quantitative data were expressed as the means ± SD. Statistical analyses were conducted utilizing GraphPad Prism 10 (GraphPad Software, USA). An unpaired 2-tailed Student’s *t* test was used for comparisons between groups, while analysis of variance (ANOVA) followed by Tukey’s post hoc test was applied for comparisons among multiple groups. Each experiment included a minimum of 3 biological replicates, unless stated otherwise. Statistical significance was determined at *P < 0.05, **P < 0.01, ***P < 0.001, and ****P < 0.0001.

## Results

### Preparation and characterization of KGN@PB@CM nanozymes

**Fig. 1.**
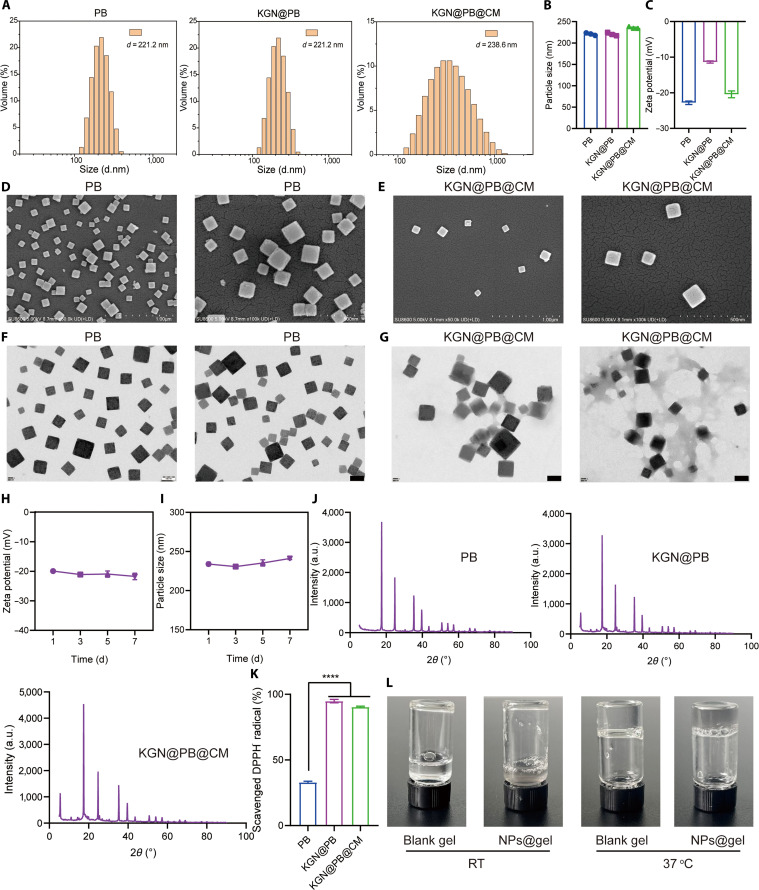
Preparation and characterization of KGN@PB@CM nanozymes. (A and B) Particle sizes of different NPs. d.nm, nanometers. (C) Zeta potentials of different NPs. (D and E) SEM images of PB and KGN@PB@CM nanozymes. (F and G) TEM images of PB and KGN@PB@CM nanozymes. (H and I) Particle size and zeta potential stability of KGN@PB@CM nanozymes. (J) XRD spectra of different NPs. a.u., arbitrary units. (K) Scavenged DPPH^.^ efficiency of different NPs. ****P < 0.0001. (L) Photographs of blank gel and KGN@PB@CM nanozyme-loaded gel at different temperatures.

We constructed a PB nanozyme as an efficient ROS scavenger to deliver KGN, a drug that enhances osteogenic differentiation. Meanwhile, to improve the macrophage-targeting ability of PB in vivo, we extracted macrophage membranes and coated them onto the surface of PB NPs. After coating with macrophage membranes, the particle size of KGN@PB nanozymes increased significantly (Figs. 1A and B). Moreover, compared with KGN@PB nanozymes, the surface potential of KGN@PB@CM nanozymes also exhibited a notable change, confirming successful membrane encapsulation (Figs. 1C). To directly observe the morphology of KGN@PB@CM nanozymes, we performed scanning electron microscopy (SEM) and transmission electron microscopy (TEM) imaging. The results revealed that KGN@PB nanozymes exhibited a cubic shape with good dispersibility. In contrast, KGN@PB@CM nanozymes displayed a distinct outer layer of cell membrane (CM) components (Figs. 1D to G). The storage stability of nanozymes is crucial for their translational and in vivo applications. Therefore, we evaluated the size and potential stability of the nanozymes over one week. As shown in Fig. 1H and I, KGN@PB@CM nanozymes exhibited minimal fluctuations in both particle size and surface potential, demonstrating excellent stability.

Ultraviolet-visible (UV-Vis) spectroscopy revealed distinct absorption spectra for the different materials (Fig. S1). X-ray photoelectron spectroscopy analysis of PB, KGN@PB, and KGN@PB@CM confirmed the presence of Fe^2+^ ions in all 3 materials, indicating the structural stability of PB during drug loading and membrane coating (Figs. S2 to S4). X-ray diffraction (XRD) further demonstrated that the crystallinity of PB nanozymes remained intact following drug loading and membrane encapsulation (Fig. 1J). The SDS–polyacrylamide gel electrophoresis of pure CM and KGN@PB@CM showed no significant changes, indicating the successful membrane coating (Fig. S5). Moreover, 2,2-diphenyl-1-picrylhydrazyl (DPPH) assay revealed that KGN@PB and KGN@PB@CM exhibited significantly enhanced ROS scavenging ability compared to the bare PB nanozymes (Fig. 1K).

Finally, we fabricated a biocompatible HA-F127 hydrogel using a physical preparation method. Upon loading the KGN@PB@CM nanozymes into the hydrogel, the solution exhibited a noticeable darkening in color. Both the blank hydrogel and the nanozyme-loaded hydrogel remained in a sol state at room temperature (RT) but underwent rapid gelation at 37 °C, demonstrating their potential for in situ gelation in vivo to prolong the therapeutic duration of the nanozymes (Fig. 1L).

### In vitro biocompatibility of KGN@PB@CM

**Fig. 2.**
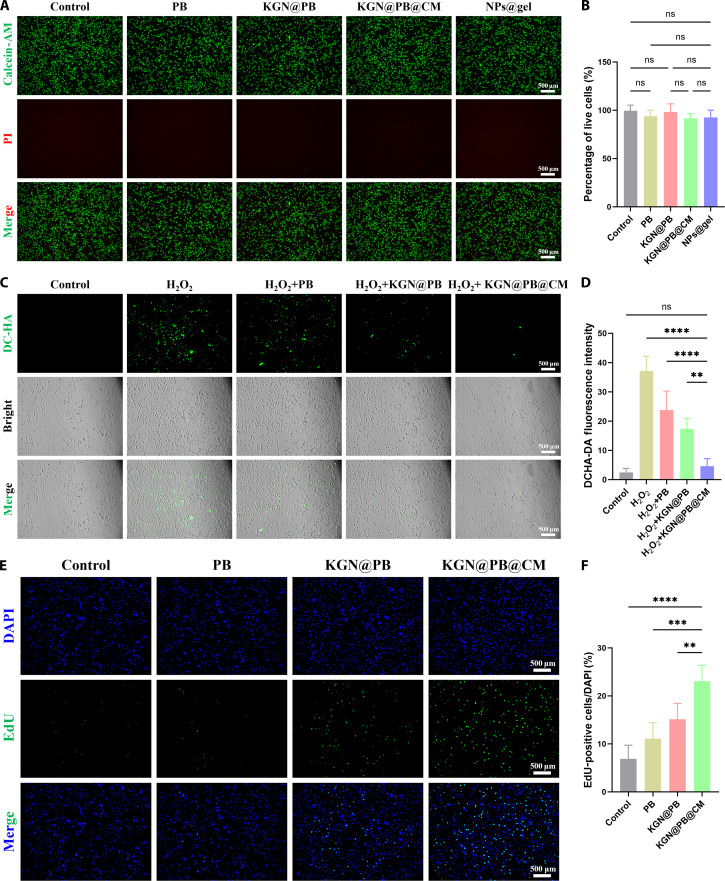
In vitro cytocompatibility, antioxidative capacity, and proliferation-promoting effects of KGN@PB@CM. (A) Representative live/dead staining images of TDSCs treated with control, PB, KGN@PB, KGN@PB@CM, and NPs@gel after 24 h. Calcein-AM (green) stains viable cells; PI (red) stains dead cells. (B) Quantification of live cell percentages among different treatment groups. No statistically significant differences were observed. (C) Representative DCFH-DA fluorescence images of TDSCs under oxidative stress (200 μM H_2_O_2_) with or without NP treatments, reflecting intracellular ROS levels. (D) Quantitative analysis of DCFH-DA fluorescence intensity. KGN@PB@CM significantly reduced ROS accumulation in comparison with other groups. (E) EdU incorporation assay showing DNA replication activity in TDSCs after 24 h of treatment. EdU (green) labels replicating nuclei; DAPI (blue) marks all nuclei. (F) Quantification of EdU-positive nuclei across groups, indicating enhanced proliferation in the KGN@PB@CM group. Data are presented as means ± SD, *n* = 5; **P < 0.01, ***P < 0.001, and ****P < 0.0001. ns, not significant.

To evaluate the cytocompatibility of the KGN@PB@CM nanoplatform, we incubated tendon-derived stem cells (TDSCs) with various formulations and measured the cell viability with calcein-AM/propidium iodide (PI) live/dead staining. As shown in Fig. 2A, all groups exhibited widespread green fluorescence with only a few red-stained cells, showing a high proportion of viable cells. Quantitative analysis revealed no significant discrepancy in the percentage of live cells among subgroups (Fig. 2B), confirming that the KGN@PB@CM nanocomposite exhibits negligible cytotoxicity toward TDSCs.

Given the critical role of oxidative stress in tendon pathophysiology, we next investigated the antioxidant capacity of KGN@PB@CM under oxidative challenge. Upon treatment with 200 μM hydrogen peroxide (H_2_O_2_), TDSCs showed robust intracellular ROS accumulation, evidenced by strong green fluorescence in 2′,7′-dichlorodihydrofluorescein diacetate (DCFH-DA) staining. Notably, cells treated with KGN@PB@CM exhibited substantially reduced fluorescence intensity compared to other formulations (Figs. 2C and D), indicating efficient ROS scavenging and superior antioxidative performance, likely attributed to the intrinsic enzymatic activity of PB nanozymes.

To further assess the influence of KGN@PB@CM on TDSC proliferation, we performed Cell Counting Kit-8 (CCK-8) assays and 5-ethynyl-2′-deoxyuridine (EdU) incorporation assays. As depicted in Fig. S6, no statistically significant discrepancy was found in optical density values between the KGN@PB@CM group and the control group at 24, 48, and 72 h, indicating that the material does not impair cell proliferation. However, the EdU staining assay (Fig. 2E) revealed a marked increase in EdU-positive nuclei in the KGN@PB@CM group compared to other groups. Quantification analysis (Fig. 2F) confirmed that KGN@PB@CM significantly enhanced DNA replication activity, suggesting a superior proliferation-supportive microenvironment.

Collectively, these data demonstrate that KGN@PB@CM exhibits excellent in vitro cytocompatibility, effective antioxidative protection, and proproliferative properties for TDSCs. These features highlight its therapeutic promise for tendon regeneration by maintaining cellular viability, mitigating oxidative damage, and enhancing reparative cell responses.

### In vitro regulation of macrophage polarization by KGN@PB@CM

To further elucidate the immunomodulatory effects of KGN@PB@CM on macrophage homeostasis, we established experimental groups, encompassing the control, lipopolysaccharide (LPS) (to simulate an inflammatory environment), PB+LPS, KGN@PB+LPS, and KGN@PB@CM+LPS groups.

**Fig. 3.**
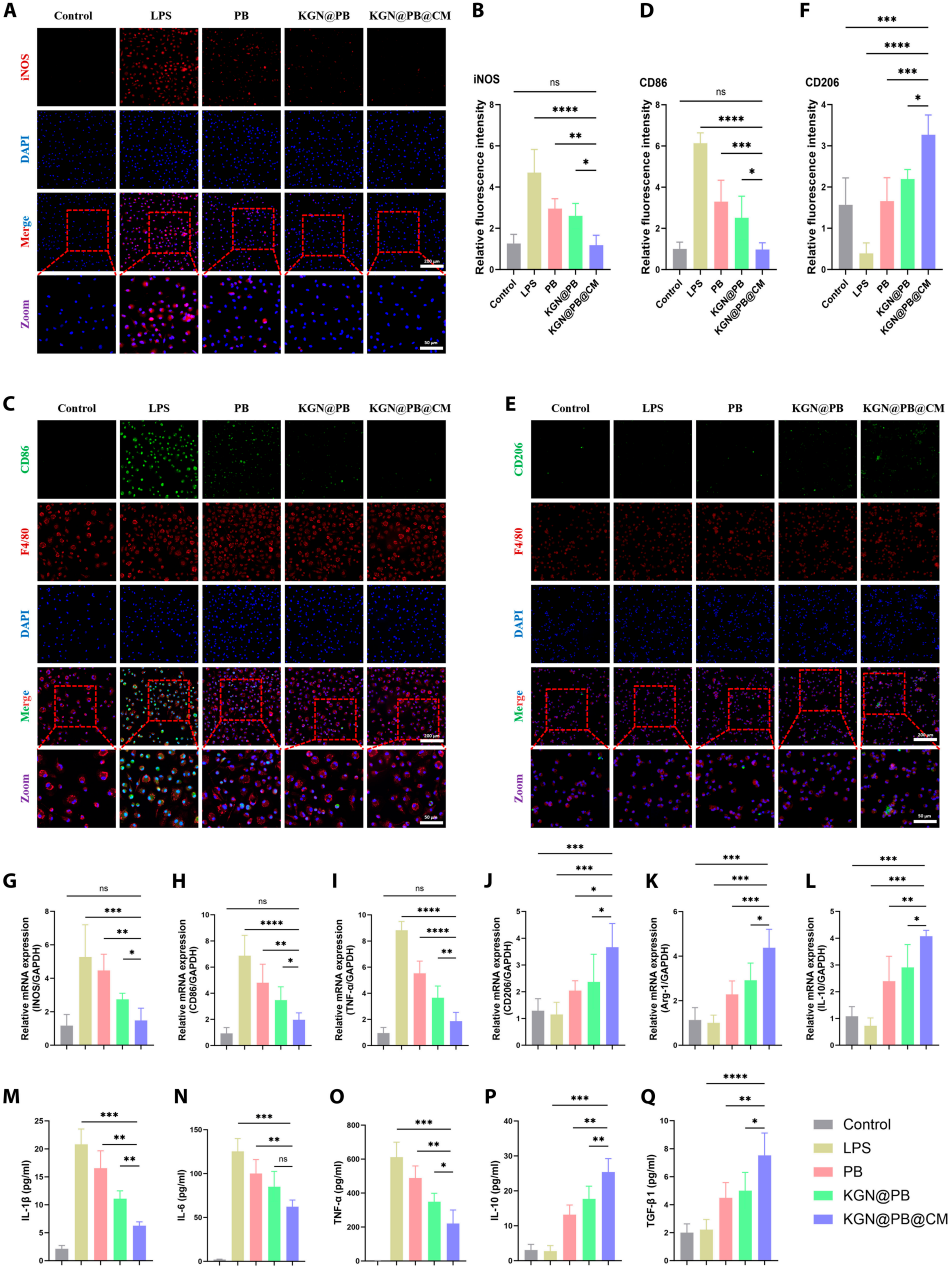
KGN@PB@CM modulates macrophage polarization and restores immune homeostasis in vitro. (A) Representative immunofluorescence images showing iNOS (M1 marker, red) expression in RAW 264.7 macrophages under different treatment conditions. DAPI (blue) was used for nuclear staining. (B) Quantitative analysis of iNOS fluorescence intensity. (C) Representative dual-staining images for CD206 (M2 marker, green) and F4/80 (pan-macrophage marker, red). (D) Quantification of CD86 (M1 marker) fluorescence intensity. (E) Representative immunofluorescence images showing CD206^+^F4/80^+^ macrophages in different groups. (F) Quantitative analysis of CD206 fluorescence intensity. (G to L) RT-qPCR analysis of M1-related genes (*iNOS*, *CD86*, *TNF-α*, and *IL-6*) and M2-related genes (*Arg-1*, *CD206*, and *IL-10*) in different treatment groups. (M to Q) ELISA results showing the concentrations of inflammatory cytokines IL-1β, IL-6, TNF-α and anti-inflammatory cytokines IL-10 and TGF-β1 in macrophage supernatants. Data are presented as means ± SD, *n* = 5; *P < 0.05, **P < 0.01, ***P < 0.001, and ****P < 0.0001.

Immunofluorescence staining revealed that KGN@PB@CM effectively inhibited LPS-induced M1 polarization of macrophages, as evidenced by markedly reduced inducible nitric oxide synthase (iNOS) and CD86 expression compared with that in the LPS group (Figs. 3A to D). Conversely, KGN@PB@CM significantly enhanced M2 polarization, as indicated by an increased population of CD206^+^ macrophages (Figs. 3E and F), suggesting its potential to reprogram macrophages toward a proregenerative phenotype.

Quantitative polymerase chain reaction (qPCR) analysis further corroborated these findings. LPS treatment up-regulated proinflammatory markers such as *CD86*, *IL-6*, and *TNF-α*, while down-regulating anti-inflammatory genes including *Arg-1*, *CD206*, and *IL-10*. Treatment with KGN@PB@CM reversed this trend, significantly suppressing proinflammatory gene expression while restoring or enhancing the transcription of anti-inflammatory markers (Fig. 3G to L).

To confirm this phenotype switch at the protein level, we performed enzyme-linked immunosorbent assay (ELISA) assays. The significant reduction of interleukin-1β (IL-1β), IL-6, and tumor necrosis factor-α (TNF-α) was identified in the KGN@PB@CM group, whereas the significant elevation of IL-10 and transforming growth factor-β1 (TGF-β1) were determined in comparison to the LPS group (Figs. 3M to Q). These findings confirm that KGN@PB@CM effectively regulates macrophage function by promoting M2 polarization while suppressing M1 polarization, thereby contributing to immune homeostasis within the inflammatory microenvironment.

In summary, KGN@PB@CM alleviates macrophage-mediated inflammation and rebalances immune polarization, offering a promising immunoregulatory strategy for tissue regeneration.

### KGN@PB@CM facilitates tendon regeneration and collagen remodeling in vivo

**Fig. 4.**
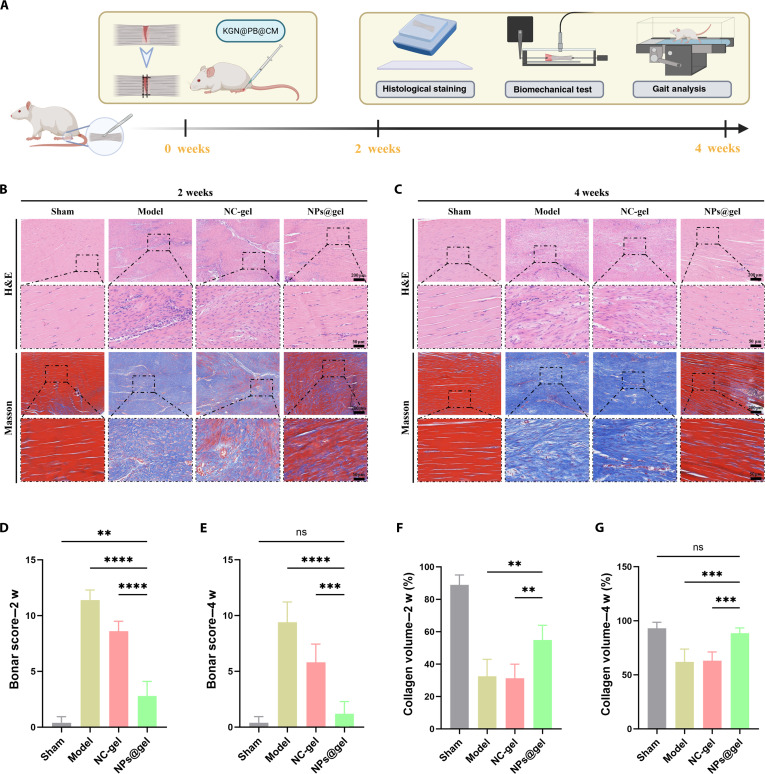
In vivo evaluation of tendon repair efficacy mediated by KGN@PB@CM hydrogel. (A) Schematic illustration of the experimental timeline and procedures for Achilles tendon injury model, including hydrogel implantation, histological analysis, gait assessment, and biomechanical testing. (B and C) Representative H&E and Masson’s trichrome staining images of tendon tissues harvested at 2 weeks (B) and 4 weeks (C) postinjury in different treatment groups: sham, model, NC-gel, and NPs@gel. KGN@PB@CM (NPs@gel) treatment group showed improved fiber alignment and enhanced collagen deposition. (D and E) Bonar histological scores at 2 and 4 weeks, respectively. Lower scores indicate better tendon tissue integrity. (F and G) Quantification of collagen volume (%) based on Masson staining at 2 and 4 weeks, respectively. KGN@PB@CM significantly increased collagen content compared to control groups. **P < 0.01, ***P < 0.001, and ****P < 0.0001.

To identify the therapeutic potential of KGN@PB@CM in vivo, we developed a rat Achilles tendon injury model and locally administered the composite hydrogel at the injury site (Fig. 4A). Animals were euthanized at 2 and 4 weeks postoperation for histological and biomechanical evaluations.

Hematoxylin and eosin (H&E) and Masson’s trichrome staining at 2 weeks revealed substantial matrix disorganization, inflammatory infiltration, and sparse collagen deposition in the untreated model group. The NPs@gel-treated group showed more aligned tissue architecture and denser collagen bundles, comparable to sham-operated controls (Fig. 4B). Bonar scoring, a semiquantitative histological grading system, showed significantly lower scores in the NPs@gel group, indicating reduced histopathological damage (Fig. 4D). This trend was maintained at 4 weeks, with further structural maturation in the NPs@gel group as evidenced by compact collagen alignment and lower Bonar scores (Figs. 4C and E).

Quantification of collagen volume via Masson staining revealed a marked increase in the NPs@gel group compared to both the model and negative control (NC)-gel groups at both 2 and 4 weeks (Figs. 4F and G), suggesting enhanced extracellular matrix (ECM) remodeling.

Collectively, these results demonstrate that KGN@PB@CM significantly promotes tendon healing by improving tissue morphology, collagen organization, and overall histological integrity in vivo.

### KGN@PB@CM enhances tenogenic differentiation of TDSCs in vitro

**Fig. 5.**
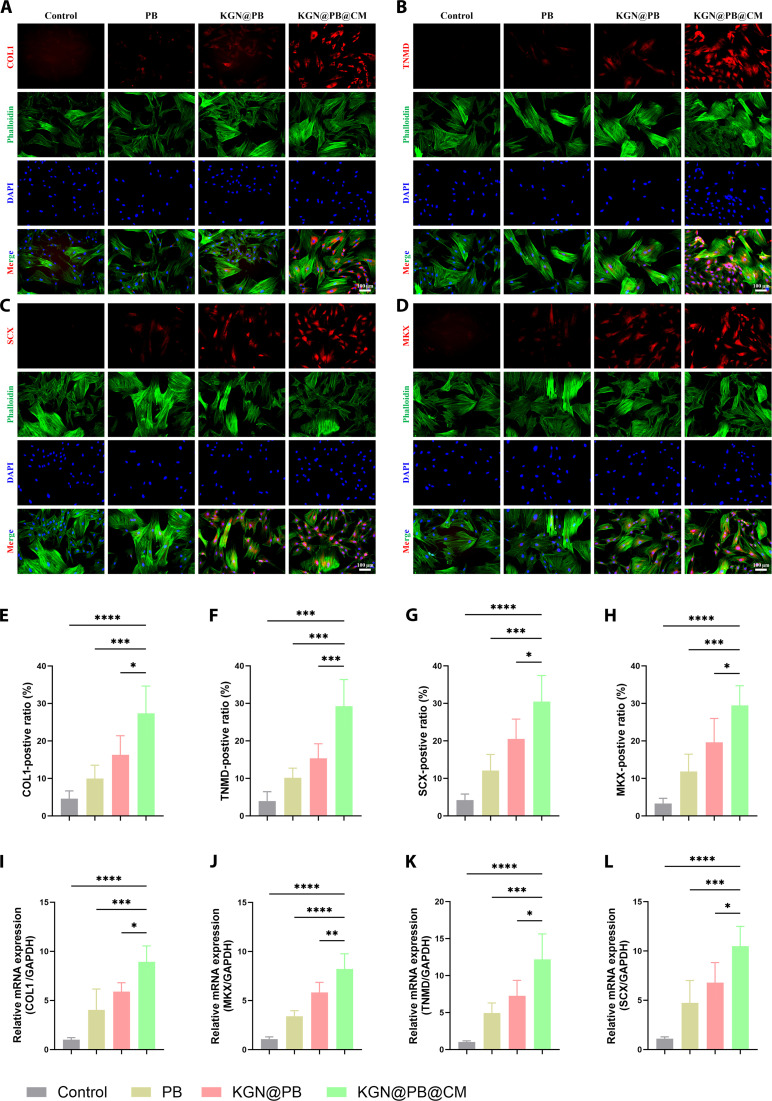
KGN@PB@CM promotes tenogenic differentiation of TDSCs in vitro. (A) Representative immunofluorescence images of COL1 expression (red) in TDSCs after 5 d of treatment with control, PB, KGN@PB, and KGN@PB@CM. Cytoskeletons were stained with phalloidin (green), and nuclei were counterstained with DAPI (blue). (B) Representative immunofluorescence images of TNMD expression (red) under different treatments, showing elevated protein expression in the KGN@PB@CM group. (C) Immunofluorescence staining of SCX (red), a transcription factor essential for tendon lineage commitment, showing increased expression in treated groups. (D) MKX (red), another tenogenic transcription factor, was markedly up-regulated upon KGN@PB@CM treatment. (E to H) Quantitative analysis of the percentage of COL1-, TNMD-, SCX-, and MKX-positive cells, respectively. (I to L) Relative mRNA expression levels of Col1, Tnmd, Mkx, and Scx in TDSCs treated with different formulations, determined by qRT-PCR (normalized to GAPDH). Data are presented as means ± SD (*n* = 3). One-way ANOVA with Tukey’s post hoc test was used for statistical analysis. *P < 0.05, **P < 0.01, ***P < 0.001, and ****P < 0.0001. Scale bar, 10 μm.

To determine the teno-inductive potential of the composite system, we evaluated the expression of tendon-specific markers in TDSCs following treatment with PB, KGN@PB, and KGN@PB@CM. Immunofluorescence staining at day 5 revealed markedly enhanced expression of key tenogenic proteins—including type I collagen (COL1), tenomodulin (TNMD), scleraxis (SCX), and Mohawk (MKX)—in the KGN@PB@CM group compared with that in the PB and KGN@PB groups (Fig. 5A to D). Quantitative analysis further confirmed a significant elevation in the percentage of marker-positive cells in the KGN@PB@CM-treated group, especially for TNMD (Fig. 5F) and SCX (Fig. 5G), both essential regulators of tendon maturation and function.

These findings were corroborated by quantitative real-time PCR (qRT-PCR) analysis, which demonstrated that KGN@PB@CM treatment significantly up-regulated the mRNA levels of Col1, Tnmd, Scx, and Mkx (Figs. 5I to L). In particular, Scx and Mkx, 2 transcription factors critical for tenocyte lineage commitment, exhibited over 2-fold elevation compared to PB and KGN@PB treatments alone. Collectively, these data suggest that KGN@PB@CM synergistically enhances tenogenic differentiation of TDSCs through both extracellular and paracrine mechanisms, likely caused by the sustained release of KGN and immunomodulatory cues from the CM component.

### KGN@PB@CM promotes tendon regeneration via in vivo regulation of M1/M2 macrophage polarization

To identify the mechanisms of which KGN@PB@CM facilitates tendon healing, we performed immunofluorescence staining on Achilles tendon tissues harvested at 2 and 4 weeks postsurgery in a rat injury model.

**Fig. 6.**
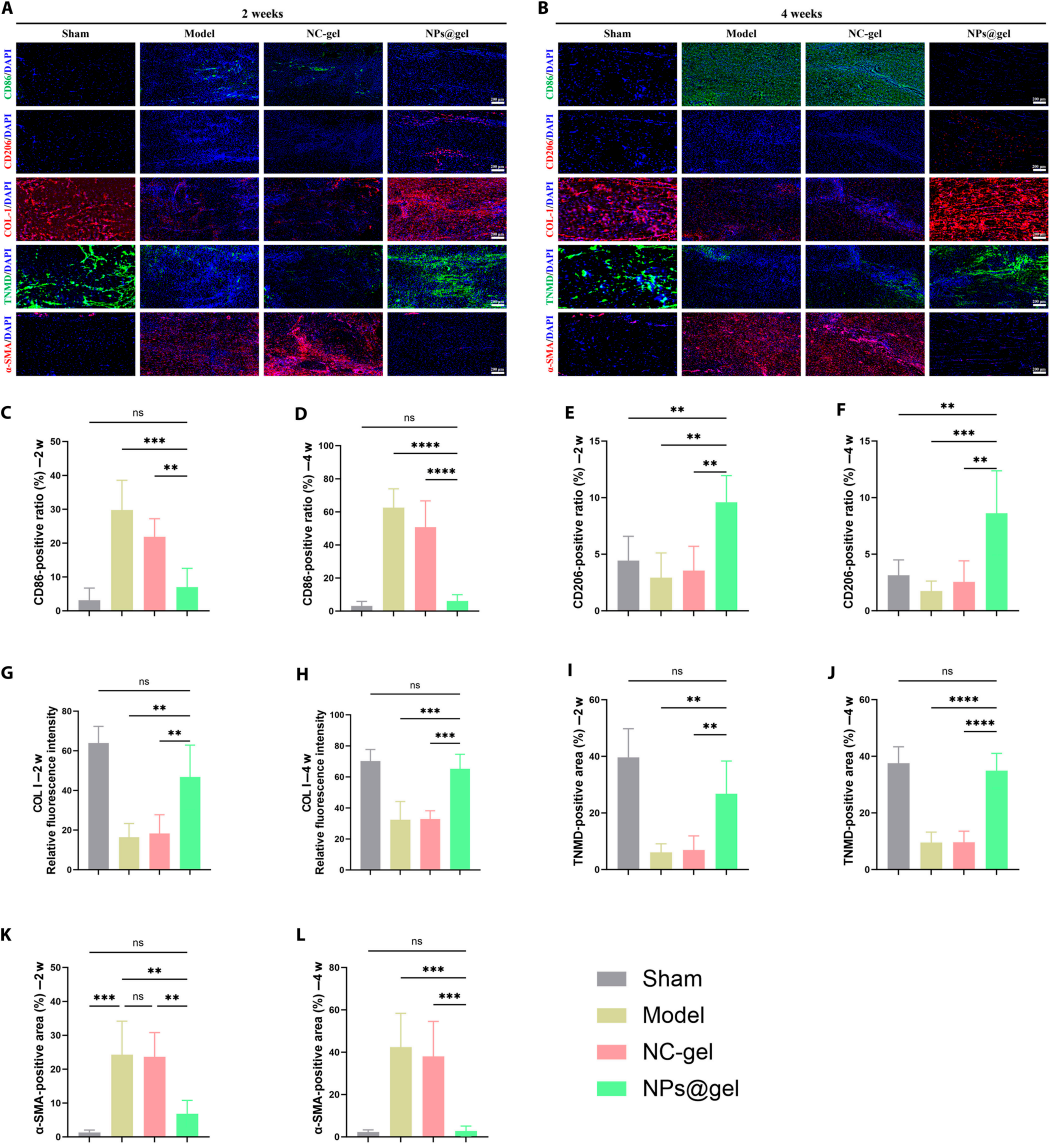
KGN@PB@CM promotes tendon healing by regulating in vivo macrophage polarization, ECM remodeling, and fibrosis suppression. (A and B) Representative immunofluorescence staining of CD86 (M1 macrophage marker), CD206 (M2 macrophage marker), TNMD, COL1, and α-SMA in Achilles tendon sections at 2 weeks (A) and 4 weeks (B) postsurgery. (C to F) Quantitative analysis of CD86^+^ and CD206^+^ cells at both time points. NPs@gel group showed significantly decreased M1 macrophage infiltration and increased M2 polarization compared to model and NC-gel groups. (G to J) Quantification of TNMD and COL1 fluorescence intensity. ECM markers were markedly up-regulated in the NPs@gel group at 2 and 4 weeks, indicating enhanced tendon matrix remodeling. (K and L) Quantification of α-SMA expression, a marker of fibrosis. NPs@gel group exhibited significantly lower α-SMA levels at both time points, suggesting reduced fibrotic response. **P < 0.01, ***P < 0.001, and ****P < 0.0001.

As exhibited in Figs. 6A to F, at 2 weeks postoperation, the NPs@gel group exhibited markedly reduced infiltration of CD86^+^ M1 macrophages compared to both the model and NC-gel groups. In contrast, the M2 macrophage marker CD206 expression was significantly elevated in the NPs@gel group, indicating a polarization shift toward a proregenerative immune profile. This trend was further accentuated at 4 weeks, with a more pronounced reduction in CD86 expression and sustained upregulation of CD206, suggesting long-term modulation of the inflammatory microenvironment by KGN@PB@CM.

In addition, immunostaining of tendon-specific ECM markers revealed enhanced regenerative outcomes in the NPs@gel group. Specifically, significantly higher expression levels of TNMD and COL1 were found in the NPs@gel group compared to the model or NC-gel groups at 2 weeks (Figs. 6G and H), and these differences became even more significant at 4 weeks (Figs. 6I and J), indicating accelerated matrix remodeling and tendon maturation.

Importantly, the fibrosis-associated marker α-smooth muscle actin (α-SMA) expression was significantly suppressed in the NPs@gel group at both time points. At 2 weeks, α-SMA expression was markedly lower than in the model and NC-gel groups, and further reduction was observed by week 4 (Figs. 6K and L), suggesting effective inhibition of fibrotic remodeling by KGN@PB@CM.

In summary, these results demonstrate that KGN@PB@CM promotes tendon regeneration through dual regulation of the immune microenvironment and matrix remodeling. By suppressing M1 macrophage activation, enhancing M2 polarization, promoting tendon-specific ECM deposition, and inhibiting fibrotic marker expression, KGN@PB@CM establishes a proregenerative niche that supports functional tissue repair.

### KGN@PB@CM enhances functional recovery and mechanical reinforcement of repaired tendons in vivo

To assess whether KGN@PB@CM could facilitate functional regeneration of Achilles tendon, we performed CatWalk gait analysis and biomechanical testing at both 2 and 4 weeks postsurgery.

**Fig. 7.**
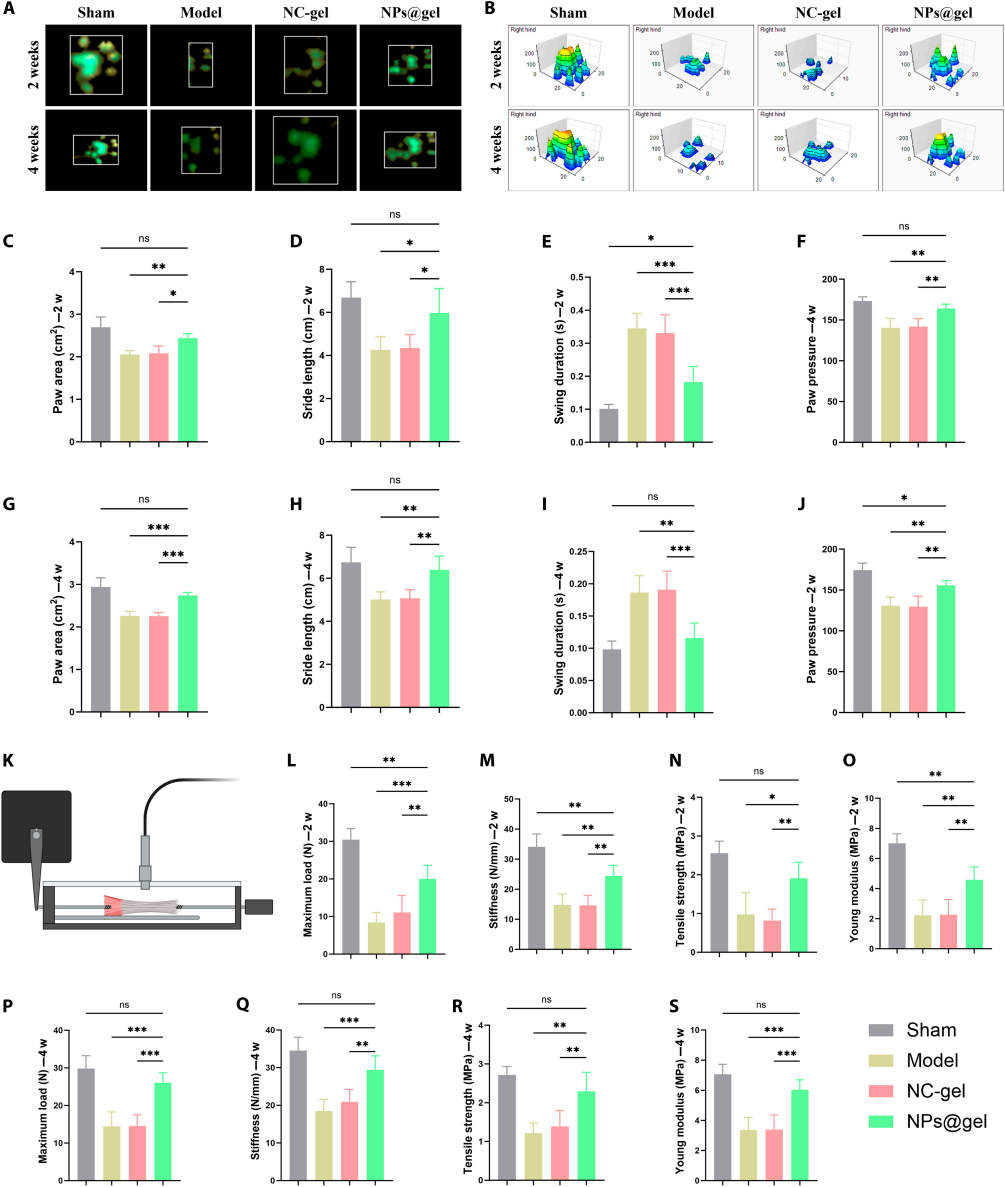
Functional and mechanical evaluation of Achilles tendon repair following KGN@PB@CM treatment. (A and B) Representative paw print heatmaps (A) and 3-dimensional pressure distribution maps (B) acquired by CatWalk XT system at 2 and 4 weeks postoperation in sham, model, NC-gel, and NPs@gel groups. (C to J) Quantification of gait parameters including paw contact area (C and G), stride length (D and H), swing duration (E and I), and paw pressure (F and J) at both 2 and 4 weeks. The NPs@gel group showed significant improvement in gait coordination over time. (K) Schematic illustration of uniaxial tensile testing for biomechanical assessment. (L to S) Biomechanical parameters of repaired tendons at 2 weeks (L to O) and 4 weeks (P to S), including maximum failure load, stiffness, tensile strength, and Young’s modulus. NPs@gel-treated tendons exhibited significantly enhanced mechanical properties compared to the control and NC-gel groups. *P < 0.05, **P < 0.01, and ***P < 0.001.

CatWalk XT system revealed that rats treated with KGN@PB@CM (NPs@gel) exhibited markedly improved locomotor coordination (Figs. 7A and B). At 2 weeks postsurgery, the NPs@gel group displayed significantly increased paw contact area and stride length, reduced swing duration, and enhanced paw pressure compared to both model and NC-gel groups (Figs. 7C to F). These improvements became even more pronounced by 4 weeks, where gait parameters in the NPs@gel group approached those of the sham group (Figs. 7G to J), indicating substantial recovery of functional tendon integrity.

To further evaluate structural recovery, uniaxial tensile testing was conducted on explanted tendons (Fig. 7K). At 2 weeks, tendons in the NPs@gel group showed higher failure load, tensile strength, stiffness, and Young’s modulus than those in both control and NC-gel groups (Figs. 7L to O). By 4 weeks, these mechanical properties were further improved (Figs. 7P to S), demonstrating that KGN@PB@CM effectively promoted biomechanical reinforcement of the healing tendon.

Collectively, these findings highlight that KGN@PB@CM not only accelerates functional gait recovery but also enhances mechanical strength of regenerated tendon tissue, underscoring its translational potential for tendon repair applications.

### In vivo biocompatibility evaluation of KGN@PB@CM

To ensure biosafety for in vivo applications, we performed the histological evaluation of major organs 8 weeks postimplantation. H&E and Masson’s trichrome staining were applied to evaluate the morphology of the heart, lung, spleen, liver, and kidney across sham, model, NC-gel, and NPs@gel groups (Fig. S7). No evidence of inflammatory cell infiltration, necrosis, fibrotic lesions, or tissue degeneration was observed in any group. Furthermore, Masson staining showed no excessive collagen deposition or fibrosis in any of the examined organs. In addition, there was no significant difference in body weight between mice. Collectively, these results confirm that the implanted hydrogel formulations exhibit excellent long-term biocompatibility and do not induce systemic toxicity or organ damage in vivo.

## Discussion

The prevalence of tendon injuries presents a significant challenge within the fields of orthopedics and sports medicine, particularly due to the tendons’ limited capacity for self-repair and the often inadequate functional recovery postinjury [20]. Tendons, which serve as critical connective tissues linking muscle to bone, frequently sustain injuries through overuse or acute trauma, resulting under conditions such as tendinopathy and ruptures [21,22]. The healing cascade of tendons is characterized by its inherently slowness and inefficiency, often inducing the generation of fibrous scar tissue that lacks the biomechanical properties of the original tendon tissue, thus jeopardizing overall functional recovery and patient outcomes [23,24]. Current therapeutic interventions, including surgical repairs and rehabilitation, have not demonstrated sufficient efficacy in restoring tendon integrity, highlighting an urgent need for innovative treatment strategies that enhance tendon regeneration and functional recovery [25].

This study investigates the development of KGN@PB@CM nanozymes, which are designed to target the dual challenges of oxidative stress and inflammation in TDSCs during the healing process. By integrating advanced nanotechnology with biological principles, the research aims to elucidate the underlying mechanisms by which KGN@PB@CM modulates macrophage polarization and enhances TDSC viability under oxidative stress conditions. The promising results indicate that these nanozymes not only improve the cellular environment for tendon regeneration but also facilitate the polarization of macrophages toward a proregenerative phenotype, thereby offering a novel therapeutic approach for tendon injuries.

In this study, we have demonstrated that KGN@PB@CM nanozymes significantly enhance tendon regeneration through several molecular mechanisms, particularly by modulating macrophage polarization and mitigating oxidative stress. The results indicate that KGN@PB@CM not only inhibits the M1 polarization of macrophages—associated with proinflammatory responses—but also promotes the polarization toward the M2 phenotype, known to facilitate tissue repair and regeneration. This shift was evidenced by decreased expression of M1 markers iNOS and CD86 and elevated expression of M2 markers CD206 and IL-10, highlighting the potential of KGN@PB@CM in reprogramming the inflammatory microenvironment to a proregenerative state. These results align with published studies that emphasize the critical role of macrophage polarization in tissue healing processes and indicate that targeting macrophage behavior may serve as a viable treatment strategy for enhancing tendon repair mechanisms [26,27].

Furthermore, the antioxidant properties of KGN@PB@CM nanozymes play a crucial role in protecting TDSCs from oxidative stress, which is detrimental to cellular function and survival. The DPPH assay and subsequent fluorescence microscopy confirmed that KGN@PB@CM significantly reduced ROS levels within TDSCs exposed to oxidative conditions. By alleviating oxidative damage, KGN@PB@CM could preserve cell viability and promote a favorable microenvironment for TDSC proliferation and differentiation. This is particularly relevant given the established correlation between oxidative stress and impaired healing responses in tendons, and the results underscore the potential applications of KGN@PB@CM in treating oxidative-stress-related conditions in regenerative medicine [28,29].

Last, the in vivo findings from the rat Achilles tendon injury model further demonstrate the efficacy of KGN@PB@CM in enhancing biomechanical properties and promoting functional recovery postinjury. The significant improvements in load-bearing capacity and tensile strength of the repaired tendons indicate that KGN@PB@CM not only fosters biological healing but also restores mechanical integrity, which is crucial for the overall recovery of tendon function [30,31]. This highlights the dual role of KGN@PB@CM in both biological and mechanical aspects of tendon healing, reinforcing its potential as a comprehensive therapeutic approach for tendon injuries. Future explorations should continue to determine the long-term effects of such therapies and their implications for clinical applications in tendon repair and rehabilitation.

The limitations warrant careful consideration for this work. First, while the use of a rat model provides valuable insights into the therapeutic potential of KGN@PB@CM, the translational applicability to human subjects remains uncertain. The relatively small sample sizes in some experimental groups may also affect the statistical robustness of the findings, potentially limiting the generalizability of the findings. In addition, the absence of long-term follow-up data on functional recovery and tissue remodeling raises questions about the durability of the observed benefits. Future research should aim to incorporate larger animal models, extended observation periods, and clinical trials to fully elucidate the long-term efficacy and safety profile of KGN@PB@CM in tendon regeneration.

In conclusion, current study investigates the promising therapeutic potential of KGN@PB@CM in promoting tendon regeneration through multifaceted mechanisms, including modulation of macrophage polarization, enhancement of antioxidant activity, and improvement of biomechanical properties. The findings suggest that KGN@PB@CM not only fosters a proregenerative microenvironment but also facilitates functional recovery and mechanical reinforcement of injured tendons. These outcomes identify its potential as a novel treatment strategy for tendon injuries and warrant further investigation to explore its applicability in broader regenerative medicine contexts.

## Data Availability

The data used to support the findings of this study are available from the corresponding author upon request.

## References

[1] Lyu K, Liu T, Chen Y, Lu J, Jiang L, Liu X, Liu X, Li Y, Li S. A “cell-free treatment” for tendon injuries: Adipose stem cell-derived exosomes. Eur J Med Res. 2022;27:75. 35643543 10.1186/s40001-022-00707-x PMC9148514

[2] Jiang L, Lu J, Chen Y, Lyu K, Long L, Wang X, Liu T, Li S. Mesenchymal stem cells: An efficient cell therapy for tendon repair (review). Int J Mol Med. 2023;52(2):70. 37387410 10.3892/ijmm.2023.5273 PMC10373123

[3] Hou J, Yang R, Vuong I, Li F, Kong J, Mao H-Q. Biomaterials strategies to balance inflammation and tenogenesis for tendon repair. Acta Biomater. 2021;130:1–6. 34082095 10.1016/j.actbio.2021.05.043

[4] Alvarez-Nemegyei J, Canoso JJ. Evidence-based soft tissue rheumatology IV: Anserine bursitis. J Clin Rheumatol. 2004;10(4):205–256. 17043509 10.1097/01.rhu.0000135561.41660.b0

[5] Wan R, Luo Z, Nie X, Feng X, He Y, Li F, Liu S, Chen W, Qi B, Qin H, et al. A mesoporous silica-loaded multi-functional hydrogel enhanced tendon healing via immunomodulatory and pro-regenerative effects. Adv Healthc Mater. 2024;13(26): Article 2400968. 10.1002/adhm.202400968 38591103

[6] Lin J, Zhou W, Han S, Bunpetch V, Zhao K, Liu C, Yin Z, Ouyang H. Cell-material interactions in tendon tissue engineering. Acta Biomater. 2018;70:1–11. 29355716 10.1016/j.actbio.2018.01.012

[7] Russo V, Prencipe G, Mauro A, El Khatib M, Haidar-Montes AA, Cambise N, Turriani M, Stöckl J, Steinberger P, Lancia L, et al. Assessing the functional potential of conditioned media derived from amniotic epithelial stem cells engineered on 3D biomimetic scaffolds: An in vitro model for tendon regeneration. Mater Today Bio. 2024;25: Article 101001. 10.1016/j.mtbio.2024.101001 PMC10899023 38420144

[8] Ye J, Li Q, Zhang Y, Su Q, Feng Z, Huang P, Zhang C, Zhai Y, Wang W. ROS scavenging and immunoregulative EGCG@cerium complex loaded in antibacterial polyethylene glycol-chitosan hydrogel dressing for skin wound healing. Acta Biomater. 2023;166:155–166. 37230435 10.1016/j.actbio.2023.05.027

[9] Zhang X, Fan H, Su L, Wang Y, Chen G. Mdivi-1 attenuates sepsis-associated acute lung injury by inhibiting M1 alveolar macrophage polarization and Pyroptosis. Mediat Inflamm. 2025;2025: Article 3675276. 10.1155/mi/3675276 PMC11972854 40196168

[10] Zhang B, Yang Y, Yi J, Zhao Z, Ye R. Hyperglycemia modulates M1/M2 macrophage polarization via reactive oxygen species overproduction in ligature-induced periodontitis. J Periodontal Res. 2021;56(5):991–1005. 34190354 10.1111/jre.12912

[11] Cui J, Ning LJ, Wu FP, Hu RN, Li X, He SK, Zhang YJ, Luo JJ, Luo JC, Qin TW. Biomechanically and biochemically functional scaffold for recruitment of endogenous stem cells to promote tendon regeneration. npj Regener Med. 2022;7(1):26. 10.1038/s41536-022-00220-z PMC9043181 35474221

[12] Xu W, Wang Y, Liu E, Sun Y, Luo Z, Xu Z, Liu W, Zhong L, Lv Y, Wang A, et al. Human iPSC-derived neural crest stem cells promote tendon repair in a rat patellar tendon window defect model. Tissue Eng Part A. 2013;19(21–23):2439–2451. 23815150 10.1089/ten.tea.2012.0453 PMC3807699

[13] Tarafder S, Brito JA, Minhas S, Effiong L, Thomopoulos S, Lee CH. In situ tissue engineering of the tendon-to-bone interface by endogenous stem/progenitor cells. Biofabrication. 2019;12(1):15008. 10.1088/1758-5090/ab48ca PMC6904927 31561236

[14] Zhu L, Yang Y, Li X, Zheng Y, Li Z, Chen H, Gao Y. Facile preparation of indocyanine green and tiny gold nanoclusters co-loaded nanocapsules for targeted synergistic sono-/photo-therapy. J Colloid Interface Sci. 2022;627:596–609. 35872417 10.1016/j.jcis.2022.07.084

[15] Yu L, Wang Z, Mo Z, Zou B, Yang Y, Sun R, Ma W, Yu M, Zhang S, Yu Z. Synergetic delivery of triptolide and Ce6 with light-activatable liposomes for efficient hepatocellular carcinoma therapy. Acta Pharm Sin B. 2021;11(7):2004–2015. 34386334 10.1016/j.apsb.2021.02.001 PMC8343191

[16] Feng H, Liu E, Gao N, Qiu Y, Zhou Z, Tian H, Wang Y. Targeted reprogramming of macrophages by Nanozyme for accelerated wound healing. ACS Appl Mater Interfaces. 2025;17(25):36356–36365. 40521762 10.1021/acsami.5c03142

[17] Zhu Q, Ma Z, Li H, Wang H, He Y. Enhancement of rotator cuff tendon–bone healing using combined aligned electrospun fibrous membranes and kartogenin. RSC Adv. 2019;9(27):15582–15592. 35514830 10.1039/c8ra09849b PMC9064336

[18] Yuan X, Wan J, Yang Y, Huang L, Zhou C, Su J, Hua S, Pu H, Zou Y, Zhu H, et al. Thermosensitive hydrogel for cartilage regeneration via synergistic delivery of SDF-1α like polypeptides and kartogenin. Carbohydr Polym. 2023;304: Article 120492. 36641179 10.1016/j.carbpol.2022.120492

[19] Maffulli N, Longo UG, Franceschi F, Rabitti C, Denaro V. Movin and Bonar scores assess the same characteristics of tendon histology. Clin Orthop Relat Res. 2008;466:1605–1611. 18437501 10.1007/s11999-008-0261-0 PMC2505245

[20] Hauschild VD, Grier T, Schuh-Renner A, Forrest LJ, Hirleman CE, Pinyan E, Jones BH. Pectoralis major injuries in the military: A surveillance approach to reduce an underestimated problem. BMJ Mil Health. 2022;168(4):286–291. 10.1136/bmjmilitary-2020-001648 33547189

[21] Lu Y, Chan K, Li G, Zhang J. Tenogenic differentiation of mesenchymal stem cells and noncoding RNA: From bench to bedside. Exp Cell Res. 2016;341(2):237–242. 26724570 10.1016/j.yexcr.2015.12.014

[22] Chartier C, ElHawary H, Baradaran A, Vorstenbosch J, Xu L, Efanov JI. Tendon: Principles of healing and repair. Semin Plast Surg. 2021;35(3):211–215. 34526870 10.1055/s-0041-1731632 PMC8432990

[23] Jiang F, Zhao H, Zhang P, Bi Y, Zhang H, Sun S, Yao Y, Zhu X, Yang F, Liu Y, et al. Challenges in tendon-bone healing: Emphasizing inflammatory modulation mechanisms and treatment. Front Endocrinol. 2024;15: Article 1485876. 10.3389/fendo.2024.1485876 PMC11576169 39568806

[24] Korcari A, Buckley MR, Loiselle AE. Characterization of scar tissue biomechanics during adult murine flexor tendon healing. J Mech Behav Biomed Mater. 2022;130: Article 105192. 35339739 10.1016/j.jmbbm.2022.105192 PMC11103245

[25] Irfan SA, Ahmed S, Ashkar A, Heyes G, Khan MW, Ahsan Nawaz SM, Siddiqui AA, Mustafa H. Comparative effectiveness of weight-bearing strategies on functional recovery in acute Achilles tendon rupture: A network meta-analysis. Foot Ankle Surg. 2025;37(7):561–569. 10.1016/j.fas.2025.02.013 40057392

[26] Wang Y, Lu X, Lu J, Hernigou P, Jin F. The role of macrophage polarization in tendon healing and therapeutic strategies: Insights from animal models. Front Bioeng Biotechnol. 2024;12: Article 1366398. 38486869 10.3389/fbioe.2024.1366398 PMC10937537

[27] Luo G, Li J, Chen S, Yuan Z, Sun Z, Lou T, Chen Z, Liu H, Zhou C, Fan C, et al. Polylactic acid electrospun membranes coated with chiral hierarchical-structured hydroxyapatite nanoplates promote tendon healing based on a macrophage-homeostatic modulation strategy. Bioact Mater. 2025;47:460–480. 40034408 10.1016/j.bioactmat.2025.01.027 PMC11872693

[28] Jeong Y, Yang D, Solidum JG, Ortinau L, Park D. Comparative single-cell analysis reveals tendon progenitor dysfunction by age-associated oxidative stress and its restoration by antioxidant treatments. J Cell Physiol. 2025;240(2): Article e70016. 39987523 10.1002/jcp.70016 PMC13151180

[29] Fu S, Yeung M, Rolf CG, Yung PS, Chan K, Hung L. Hydrogen peroxide induced tendinopathic changes in a rat model of patellar tendon injury. J Orthop Res. 2018;36(12):3268–3274. 30066401 10.1002/jor.24119

[30] Landis WJ, Silver FH. The structure and function of normally mineralizing avian tendons. Comp Biochem Physiol A Mol Integr Physiol. 2002;133(4):1135–1157. 12485697 10.1016/s1095-6433(02)00248-9

[31] Enwemeka CS. Functional loading augments the initial tensile strength and energy absorption capacity of regenerating rabbit Achilles tendons. Am J Phys Med Rehabil. 1992;71(1):31–38. 1739442 10.1097/00002060-199202000-00008

