# Supplementary material for: Bioinspired Nanocomposite for Targeted Immunoengineering and Improved Tendon Regeneration
Source: Cyborg Bionic Syst. 2026 Apr 23;7:0503. doi: 10.34133/cbsystems.0503 (PMC13103462; doi:10.34133/cbsystems.0503)
Supplement: Supplementary 1 — Figs. S1 to S7 [file cbsystems.0503.f1.docx]

**Supporting information**


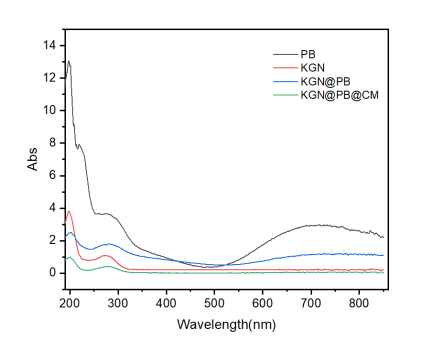


**Figure S1**. The UV-Vis spectra of different materials.


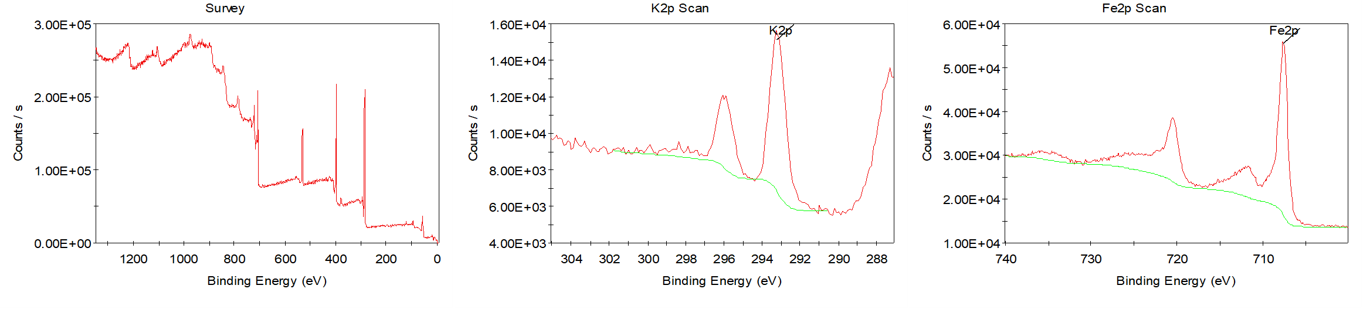


**Figure S2**. The XPS spectra of PB.


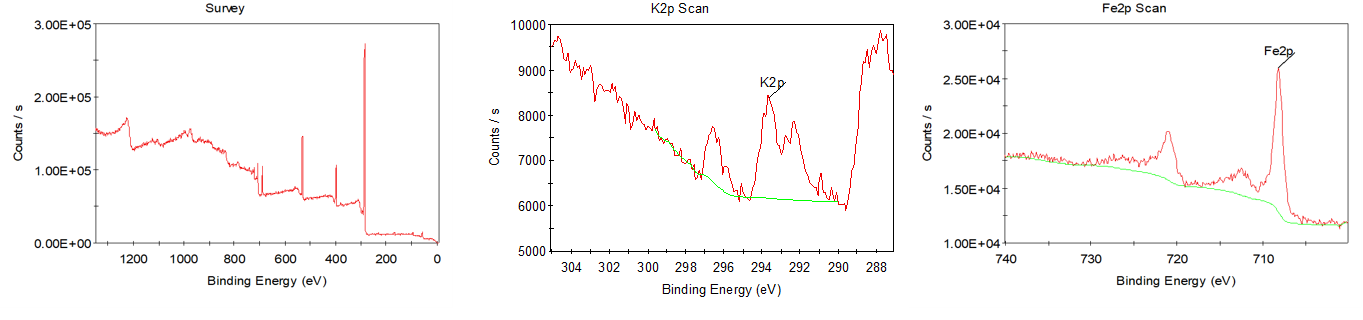


**Figure S3**. The XPS spectra of KGN@PB.


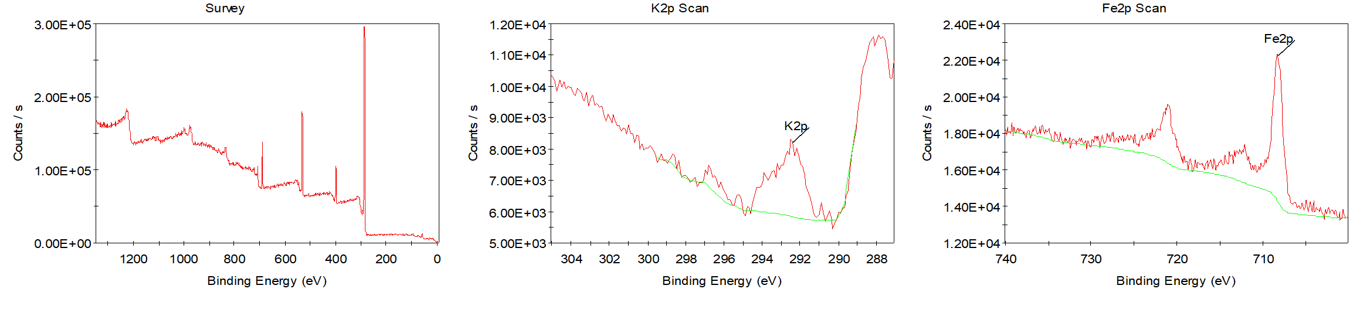


**Figure S4**. The XPS spectra of KGN@PB@CM.


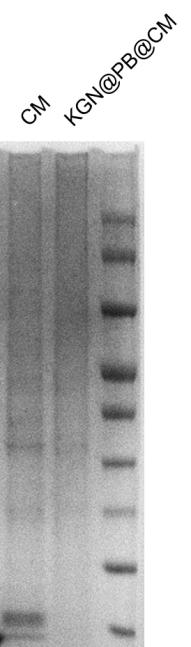


**Figure S5**. The SDS-PAGE of free CM and KGN@PB@CM.


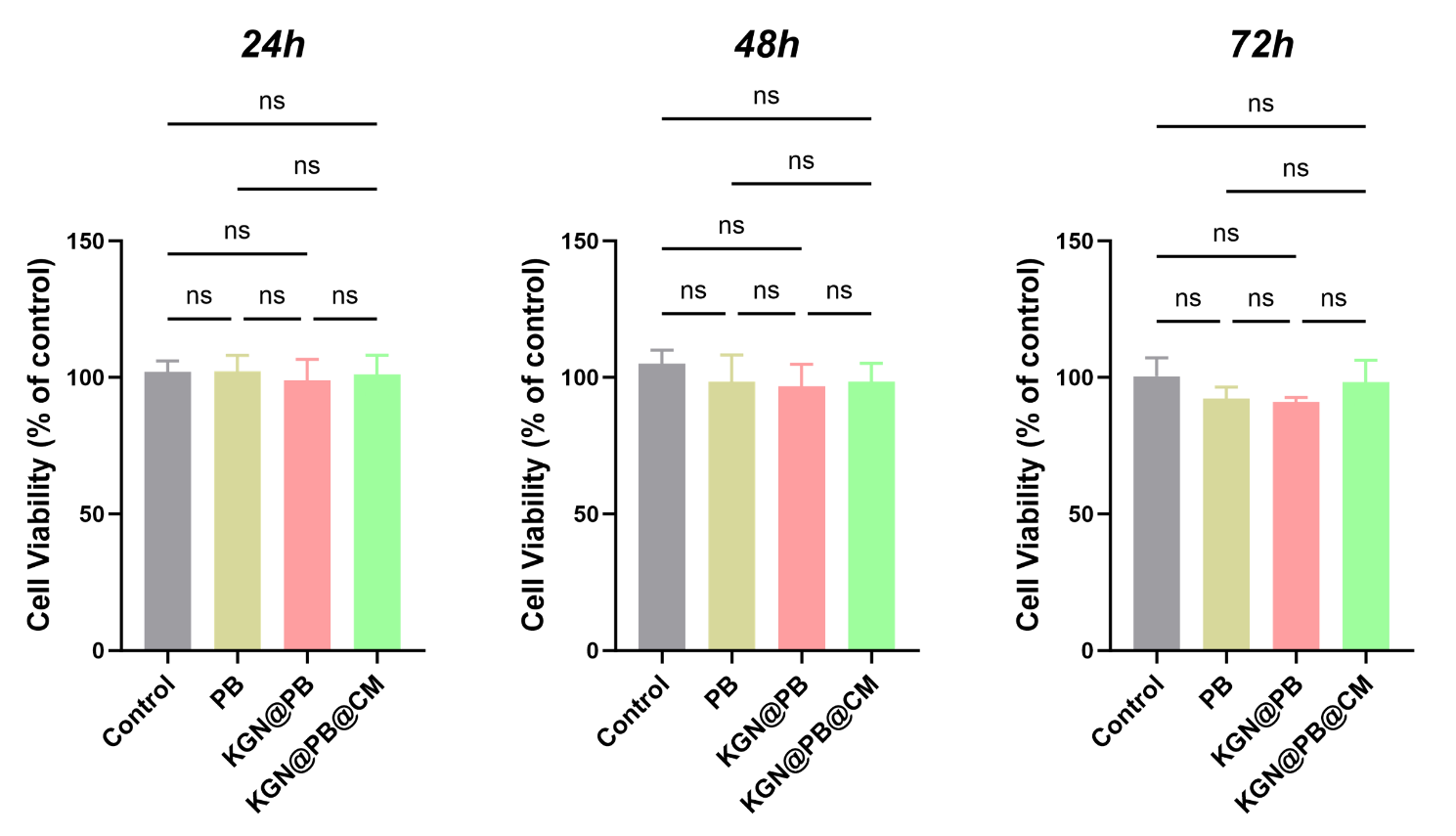


**Figure S6. Cell viability of TDSCs following treatment with PB, KGN@PB, and KGN@PB@CM for 24, 48, and 72 h assessed by CCK-8 assay.**

No significant differences in cell viability were observed at any timepoint compared to the control group, confirming the biosafety of all tested formulations.

Data are presented as mean ± SD, n = 3; ns = not significant.


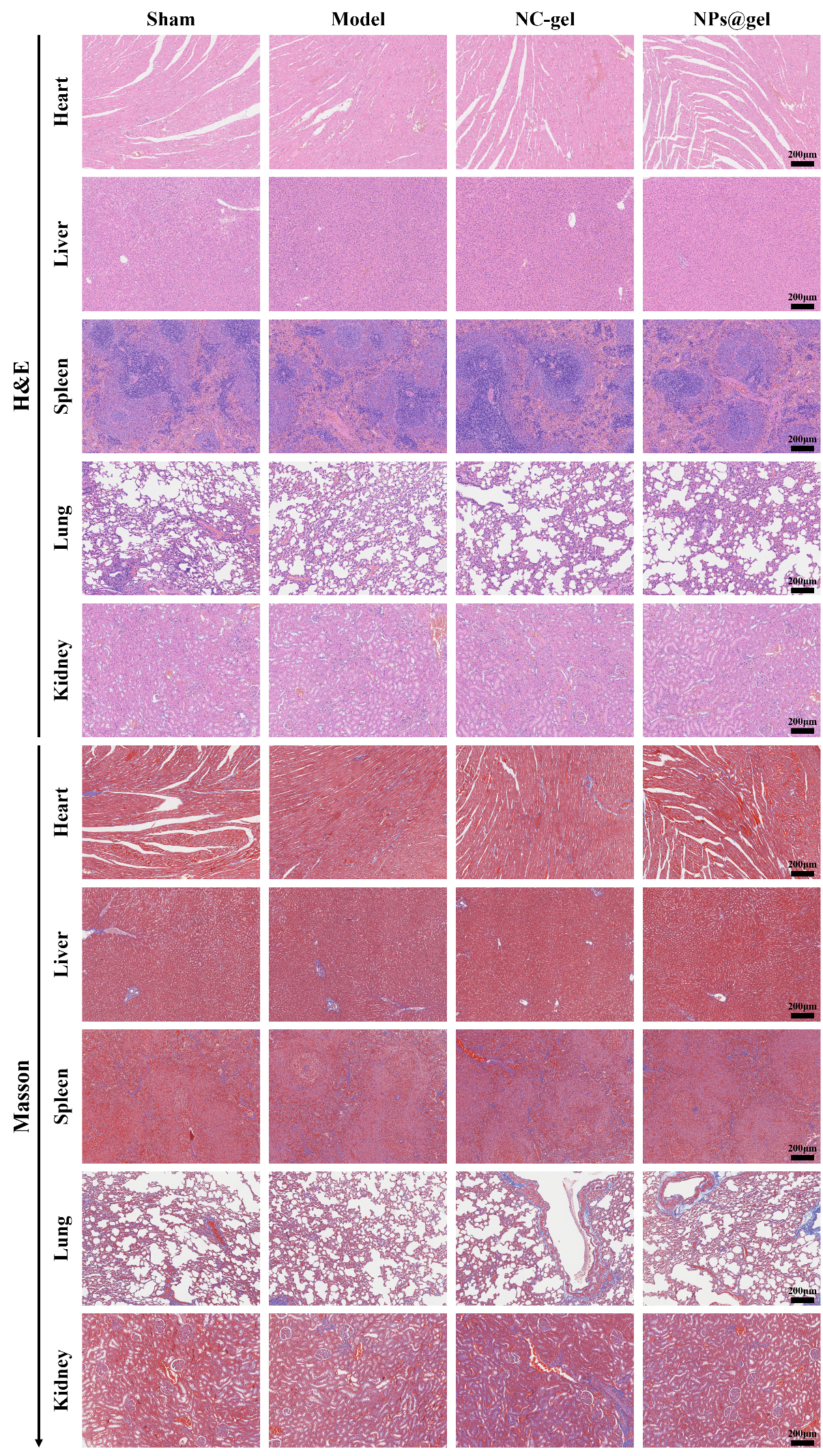


**Figure S7.** *In vivo biocompatibility assessment of different treatments at 8 weeks post-implantation.* Representative histological images of major organs (heart, liver, spleen, lung, and kidney) stained with hematoxylin and eosin (H&E) and Masson’s trichrome across the Sham, Model, NC-gel, and NPs@gel groups. No pathological abnormalities, such as inflammatory infiltration, necrosis, or fibrotic tissue accumulation, were observed in any group, indicating favorable systemic biocompatibility of the implanted materials. Scale bar: 200 μm.
